# Characterising B cell numbers and memory B cells in HIV infected and uninfected Malawian adults

**DOI:** 10.1186/1471-2334-10-280

**Published:** 2010-09-22

**Authors:** Herbert Longwe, Stephen Gordon, Rose Malamba, Neil French

**Affiliations:** 1 Malawi-Liverpool-Wellcome Trust Clinical Research Programme, Blantyre, Malawi; 2 Liverpool School of Tropical Medicine, University of Liverpool, Liverpool, UK; 3 Department of Medicine, College of Medicine, University of Malawi, Blantyre, Malawi; 4 Wellcome Trust/LEPRA Karonga Prevention Study, London School of Hygiene and Tropical Medicine, Chilumba, Malawi

## Abstract

**Background:**

Untreated human immunodeficiency virus (HIV) disease disrupts B cell populations causing reduced memory and reduced naïve resting B cells leading to increases in specific co-infections and impaired responses to vaccines. To what extent antiretroviral treatment reverses these changes in an African population is uncertain.

**Methods:**

A cross-sectional study was performed. We recruited HIV-uninfected and HIV-infected Malawian adults both on and off antiretroviral therapy attending the Queen Elizabeth Central hospital in Malawi. Using flow cytometry, we enumerated B cells and characterized memory B cells and compared these measurements by the different recruitment groups.

**Results:**

Overall 64 participants were recruited - 20 HIV uninfected (HIV-), 30 HIV infected ART naïve (HIV+N) and 14 HIV-infected ART treated (HIV+T). ART treatment had been taken for a median of 33 months (Range 12-60 months). Compared to HIV- the HIV+N adults had low absolute number of naïve resting B cells (111 vs. 180 cells/μl *p *= 0.008); reduced memory B cells (27 vs. 51 cells/μl *p *= 0.0008). The HIV+T adults had B-cell numbers similar to HIV- except for memory B cells that remained significantly lower (30 vs. 51 cells/μl *p *= 0.02). In the HIV+N group we did not find an association between CD4 count and B cell numbers.

**Conclusions:**

HIV infected Malawian adults have abnormal B-cell numbers. Individuals treated with ART show a return to normal in B-cell numbers but a persistent deficit in the memory subset is noted. This has important implications for long term susceptibility to co-infections and should be evaluated further in a larger cohort study.

## Background

Untreated HIV infection leads to disruption of the immune system leading to an increased risk of many infections and in particular pneumococcal disease [1,2]. A classic feature of HIV-disease progression is the consistent destruction of lymph nodes (LN) contributing to the progressive loss of the CD4 T-cells and degeneration of germinal centres. In addition there is massive and progressive destruction of B cells as a consequence of LN destruction and by a direct effect of HIV leading to apoptosis of B cells [3,4]. This decline in the B cell numbers is a major factor rendering HIV-infected individuals incapable of mounting an effective functional antibody response to pneumococcal polysaccharides [5]

In addition to increased immunoglobulin secretion and a decrease in number of naïve resting B cells [6], HIV leads to multiple measurable defects in the B-cell subpopulations. Previous reports have shown reduced numbers of circulating CD19^+^CD27^+ ^memory B cells both in adult and paediatric groups [7,8]. HIV also leads to B cell hyperactivation which is characterized by increased expression of activation markers [9,10], poor expression of CD19^+^CD21^+ ^(naive mature B cells) on peripheral B cells [11] and have increased frequency of immature transitional B cells (CD10^+^CD21^low^CD27^-^) in the peripheral blood [12].

It is now becoming clear that many of these B cell defects associated with HIV are reversed by anti retroviral therapy (ART) with the exception of memory B cells which remain low despite control of HIV load [13-16]. However despite these advances in our understanding of B cell defects associated with HIV and their reverse caused by ART, very few studies looking at these B cell abnormalities in HIV have been conducted in Africa. Ethnic origin and thus genetic background of individuals, different clades of the HIV virus, altered clinical presentation, different co-infections, and limited or delayed access to health care may result in different outcomes compared to studies from developed countries. Only one study pre ART conducted in Central Africa where they characterised peripheral B cell compartments in HIV infected individuals and observed germinal center B cells in blood has been conducted so far [17]. Treatment with antiretroviral therapy is improving the outcome of HIV-infected Africans. However they remain at increased risk for many opportunistic infections including pneumococcal disease. How far the immune system reconstitutes and to what extent B-cell defects return to normal is important for understanding whether and how vaccines for pneumococcal disease control can be used. There is limited data on the extent to which B-cell characteristics revert back to normal takes place in Africa.

We have measured B cell numbers and memory B cells numbers and relative percentages in adults in Blantyre, Malawi to see how far previously described HIV-associated abnormalities are present in our population of adults and how these abnormalities are changed after anti-retroviral therapy. This work was undertaken as part of a wider investigation of the cellular response to pneumococcal polysaccharides, results of which will be reported separately.

## Methods

### Study participants

Participants for the study were identified through the Wellcome Trust adult clinical research clinic at Queen Elizabeth Central Hospital (QECH) in Blantyre. The study aimed to recruit a total of 60 adult participants in 5 groups: 20 HIV-uninfected; 10 HIV-infected with CD4 > 200 cells/mm^3^; 10 HIV-infected with CD4 ≤ 200 cells/mm^3^;10 HIV-infected adults on ART treatment for more than one year; and 10 HIV-infected adults convalescing from an invasive pneumococcal (IPD) event. Participants were recruited into a study investigating pneumococcal polysaccharide directed B-cell responses and no formal sample size calculation was undertaken. With the exception of the IPD cases, participants were recruited after volunteering following a public advertisement in the hospital. Individuals recovering from pneumococcal disease were identified from blood culture records and approached by a study team member for consent. Study participants underwent a structured clinical assessment and then provided 5 mls of blood in EDTA for phenotypic analysis. HIV-infected participants found to have low CD4 counts and not on ART were counselled and encouraged to attend the ART clinic.

All participants gave signed informed consent before recruitment. The study was approved by the College of Medicine Research and Ethics Committee, Malawi under protocol number P 99/00/101.

### Blood specimen

Blood samples were processed within two hours of collection. CD4^+ ^T cell count and full blood count to determine the absolute lymphocyte count and the white cell count were performed on a FACSCount™ system (Becton Dickinson, San Jose, California, USA) and a Coulter^® ^HmX haematology analyser (Beckman Coulter ™ Company, Miami Florida, USA) respectively.

### B cell Immunophenotyping

The absolute counts and the relative percentages of B cells and memory B cell subpopulation (CD27^+^) were investigated. The B cells and memory B cell subset were stained with the following monoclonal antibodies conjugated with fluorescein isothiocyanate (FITC), phycoerythrin (PE), and allophycocyanin (APC) suitable for three-colour flow cytometry. The following phenotypic markers were used: CD19-FITC, CD19-PE, CD19-APC (B cell markers), CD45-FITC, CD45-APC (leukocyte markers) CD3-APC (T cell marker), CD27-PE (memory B cell maker), (All from Becton Dickinson Biosciences, San Jose, CA). Immunoglobulin isotype-matched FITC-, PE-, APC-conjugated monoclonal antibodies (Becton Dickinson Biosciences, San Jose, CA) were used as negative controls to check for non specific staining. Briefly, the red blood cells were lysed for 20 minutes using BD lyse solution (Becton Dickinson Biosciences, San Jose California, USA). The cells were washed twice with 1% faetal calf serum (FCS) in phosphate buffered saline (PBS). In between washes the cells were blocked for 15 minutes with 2 ml PBS/1%FSC/5 μg human polyclonal IgG (Sigma)/10 μl mouse polyclonal IgG (Caltag)/50 μl Immune human serum. The white blood cells were stained with the appropriate monoclonal antibody for 30 minutes in the dark. Afterwards, the cells were washed in PBS containing 1% foetal calf serum and fixed in 2% paraformaldehyde before analysis. The B cell subsets were analysed on FACSCalibur flow cytometer (Becton Dickinson Immunocytometry Systems, San Jose California, USA) with CellQuest-Pro™ software (Becton Dickinson, San Jose California, USA) used for analysis.

Lymphocytes were gated on forward and side scatter and 50, 000 events were gathered. CD19^+ ^B cells were expressed as a percentage of the total lymphocyte count and as the absolute count of CD19^+ ^lymphocytes per 1000 μL blood. Memory B cells were defined as CD19^+^CD27^+ ^B cells.

### Statistical analysis

For statistical analysis, study participants were divided into three primary groups: HIV negative (HIV-), HIV positive ART naïve (HIV+N) and HIV positive on ART (HIV+T). Within the HIV+N, three secondary groups were also analysed namely: HIV positive with low CD4 T cell counts, HIV positive with high CD4 T cell counts and IPD cases. The data were not normally distributed (Shapiro-Wilk normality test) and two-way comparisons between the study groups were analysed by Mann-Whitney U test. Correlation between absolute B cell counts and CD4 counts in HIV positive adults ART naive and on ART was performed by Spearman's rank correlation test. The median and ranges (minimum and maximum) are used as summary statistics within the text and in the tables. *P *values of less than 0.05 were considered significant.

## Results

A total of 64 participants were recruited to the study (47% male): 20 HIV-, 30 HIV+N and 14 HIV+T. Demographic and clinical characteristics of the study participants are summarised in Table 1. All the IPD cases were HIV positive. There were more women in the HIV+T group than the other groups and age did not differ significantly between all the study groups (Table 1). In the HIV+T group the CD4+ T cells count increased significantly after a mean period of 33 months of ART (*p *< 0.0001) but remained significantly less than the HIV- group (*p *= 0.006). Results of the memory B cell subset based on cell counts were similar to those based on percentages. Absolute counts of total B cells and memory B cell subset are thus presented in table 2.

**Table 1:** Demographic and clinical characteristics of study participants, by HIV infection status, ART and IPD event

Variable	Study group				
	**HIV Negative** **(n = 20)**	**HIV+ CD4 < 200** **(n = 10)**	**HIV+ CD4≥200** **(n = 10)**	**HIV+ on ART** **(n = 14)**	**IPD cases** **(n = 10)**

Age, mean years (SD)*	34.3 (10.4]	33 (6.2)	29.1 (6.0)	42.9 (10.7)	37.2 (10.4)
Sex, female percentage	35	30	60	79	30
CD4^+ ^T cell count at sampling, median [range]	784 [334-1122]	117 [37-196]	450 [335-574]	349 [166-838]	223 [40-558]
CD4+ T cell count pre-ART, median [range]	-	-	-	138 [20-180]	-
Period on ART, mean months	-	-	-	33	-
Total lymphocyte count, median [range]	1850 [800-3000]	1400 [800-1800]	1500 [1100-2600]	2050 [1000-3800]	1350 [600-2300]

*Age was not statistically significantly different between the study groups.

**Table 2:** Absolute counts (x10^3^cells/ml) of B cells and memory B cells from study participants by HIV infection status, ART and IPD cases

	**Study group**							
	
	**HIV negative**	**HIV positive**						
	
		**ART naive**					**On ART**	
		
**Variable**		**CD4 < 200**	**CD4≥200**	**IPD**	**All**	**P**^**1**^		**P**^**2**^
				
Absolute B cell count, median [range]	180.2 [70-427.5]	128.7 [48-208]	85.5 [46.8-196]	109.5 [45-230]	111.2 [45-230]	0.008	128 [20.8-445]	0.52
Absolute CD27^+ ^B cell count, median [range]	51 [20.8-129.5]	31.8 [10.3-76]	20.2 [10-62.2]	28.5 [5.4-60.3]	27 [5.4-76]	0.0008	30.3 [5.6-108]	0.02

P^1^: p value comparing all HIV positive (ART naïve) with HIV negative group. P^2^: p value comparing HIV positive on ART with HIV negative group.

### Absolute B cell counts

**Figure 1:**
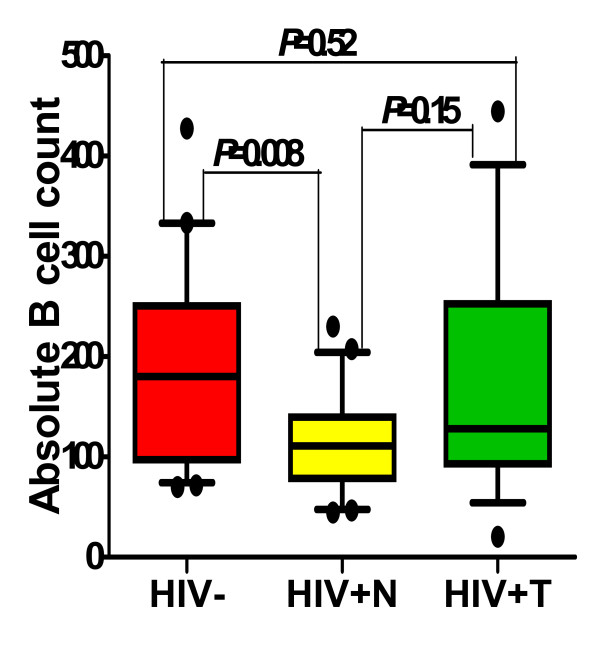
**Absolute B cell counts from HIV negative, HIV positive ART naïve and on ART**. Medians (10^th ^and 90^th ^percentiles) of absolute B cell counts (x10^3^cells/ml) in HIV negative (n = 20), HIV infected subjects ART naïve (n = 30) and HIV infected subjects on ART (n = 14).

Compared to HIV- participants-the absolute number of B cells (Figure 1) were significantly reduced in HIV+N participants (111 vs. 180 cells/μl, *p *= 0.008). The absolute B cell count was also reduced in HIV+T participants but the difference was not significant (128 cell/μl, *p *= 0.52). There were no significant differences in the median B cell counts between the HIV+N subgroups (Table 2).

### CD4 T cell counts and B cell counts in HIV-infected adults

**Figure 2:**
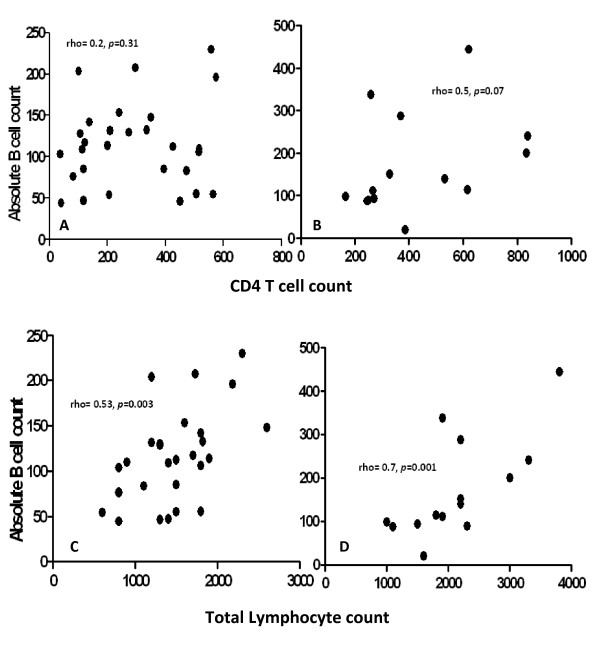
**Scatter plots evaluating correlation between CD4 T cell and absolute B cell counts and between absolute B cell counts and total lymphocyte counts in both HIV+N and HIV+T**. Scatter plots evaluating correlation between CD4 T cell counts and absolute B cell counts in HIV positive adults ART naïve (Figure 2a) and HIV positive adults on ART (Figure 2b). Correlation between absolute B cell counts and total lymphocyte counts in HIV positive adults ART naïve (Figure 2c) and HIV positive adults on ART (Figure 2d)

Overall there was no significant association between CD4 T-cell count and absolute B-cell counts in the treated and non treated subgroups. However, there was a tendency to a direct association between CD4 T cell counts and absolute B cell counts, Figure 2b, in HIV+T group, (rho = 0.5, *p *= 0.07) but not in the HIV +N participants, Figure 2a (rho = 0.2, *p *= 0.31).

### Change in absolute counts and percentages of memory B cells

Compared to HIV- participants, HIV+N participants had lower absolute counts of memory B cells (*p *= 0.0008).Similar results were also obtained for percentages of memory B cells (*p *= 0.049).

The absolute counts and percentage of the memory B cells remained low in HIV+T participants compared to HIV- participants (*p *= 0.02 and *p *= 0.005 respectively). There were no significant differences in the absolute counts and percentages of the memory B cells between the HIV+N subgroups.

## Discussion

In this small cross-sectional study, we have shown that HIV infected adults have significant reduction of total B cell numbers and memory B cells. Following ART B cell numbers are similar to HIV-uninfected adults but there are persisting defects in the memory B cell subset.

B cell numbers have been reported to be reduced during HIV infection as a result of B cell apoptosis triggered by HIV gp120 leading to B cell activation during infection [18]. As expected, it was observed that B cell numbers are significantly reduced in HIV positive adults ART naive. The counts were higher in patients on ART. These results are similar to previous report [14] which showed B cell numbers to improve with reduction of HIV viremia as a result of ART.

Memory B cells are identified by CD27 marker on the surface of the cells [19] Evaluation of memory B cells is important in determining the capacity of the immune system in producing functional and class-switched antibodies. Our findings are similar to previous studies that reported total memory B cells to be reduced (in both proportional and absolute terms) in untreated HIV infected adults [7-9,20]. A similar study which recruited African individuals also showed reduced memory B cells in HIV infected ART naïve patients [17]. Previous reports [21]showed that increased risk of pneumococcal disease in untreated HIV positive patients might partly be a consequence of depleted absolute memory B cell counts. No significant difference in the absolute memory B cell counts was detected in the IPD cases compared to HIV infected adults without IPD.

Both percentage and absolute numbers of the memory B cells did not increase in HIV positive adults on ART (mean of 33 months on ART) and were actually lower than in the ART naive HIV positive adults. These data are consistent with findings reported by De Milito *et al. *who suggested that loss of memory B cells might occur early after infection and may not be corrected by therapy. Recently a similar study that looked at activated and resting memory B cells in HIV infected individuals on ART showed an increase in numbers of resting memory B cells and a reduction in activated memory B cells [14]. However in the present study, no distinction was made between resting and activated memory B cells. A possible explanation to low numbers of memory B cells in HIV infected individuals could be that there were more activated memory B cells than resting memory B cells. However this needs to be further investigated. Some reports show ART to have no effect on susceptibility of HIV patients to IPD [21]. Our findings would be consistent with this clinical finding and may provide a partial explanation for it.

The study showed no relationship between CD4 T-cell count and B-cell numbers although in the treated HIV positive adults there appeared to be a tendency to a direct association. The failure to see an overall association may be a consequence of small study numbers. Plasma viral load, which we did not measure, may also be an important determinant of B-cell numbers and may confound any association, except in the case of the ART treated group when viral suppression allows the true relationship between B-cells and CD4 T-cells to become apparent.

The study has a number of limitations. The sample size was small, forming part of a more detailed study of antigen-specific B-cell characteristics. Selection of patients was not random and relied on volunteers responding to a public announcement. The units well known association with respiratory disease research may have inadvertently biased recruitment in favour of individuals with previous concerns about respiratory health and perhaps a population biased towards abnormal B-cell function. Nevertheless, the study points to an important failure in recovering memory B-cell subsets and a larger study is now justified, to measure the size of this problem. Viral load measurements were not made and thus we cannot conclude that the investigated participants were receiving successful suppressive ART, although all were clinically well at the time of enrolment. A prospective cohort study is now planned to investigate these findings further.

## Conclusions

The study has shown that HIV-infected Malawian adults have significant quantitative defects in B cell numbers and memory B cells. ART use is associated with normalisation of these defects but a persistent deficiency in the memory subset appears to be retained. This has important implications for long term susceptibility to co-infections and co-infection prophylaxis. Our findings are limited by the small size and cross-sectional nature and need to be evaluated further in a larger cohort study specifically designed for this purpose.

## Competing interests

The authors declare that they have no competing interests.

## Authors' contributions

HL collected, analysed the data and wrote the paper. SG supervised data analysis and critically revised the manuscript. RM recruited, vaccinated and collected the blood samples from the subjects. NF originated the idea of the research, supervised the collection and analysis of data and revised the manuscript. All authors read and approved the final manuscript.

## Pre-publication history

The pre-publication history for this paper can be accessed here:

http://www.biomedcentral.com/1471-2334/10/280/prepub
